# Association of *SLC11A1* 3′UTR (GT)_n_ Microsatellite Polymorphisms with Resistance to Paratuberculosis in Sheep

**DOI:** 10.3390/pathogens14111150

**Published:** 2025-11-12

**Authors:** Antonia Mataragka, Anastasios Klavdianos Papastathis, John Ikonomopoulos

**Affiliations:** 1 Laboratory of Anatomy and Physiology of Farm Animals, Department of Animal Science, School of Animal Biosciences, Agricultural University of Athens, 78 Iera Odos Str., GR-11855 Athens, Greece; 2 Institute for Fundamental Biomedical Research, Biomedical Sciences Research Center “Alexander Fleming”, Fleming 34, 16672 Vari, Greece

**Keywords:** *Mycobacterium avium* subsp. *paratuberculosis*, Johne’s disease, sheep, *SLC11A1* gene, microsatellite polymorphism, disease resistance

## Abstract

Paratuberculosis (Johne’s disease) is a chronic enteric infection of ruminants caused by *Mycobacterium avium* subsp. *paratuberculosis* (MAP), leading to significant economic losses in livestock production. While the *solute carrier family 11 member 1* (*SLC11A1*) gene has been implicated in resistance to intracellular pathogens in several species, its role in ovine paratuberculosis remains largely uncharacterized. The present study investigated whether polymorphic variation in the *SLC11A1* 3′ untranslated region (3′UTR) (GT)_n_ microsatellite is associated with resistance or susceptibility to MAP infection in sheep. A total of 138 sheep from three breeds (Karagouniki, Boutsika, and Chios) were genotyped. Gene expression analysis was subsequently performed on a subset of 53 animals, which comprised rigorously phenotyped MAP-resistant (n = 18) and MAP-sensitive (n = 35) individuals from the Karagouniki breed. Four predominant alleles, (GT)_21_, (GT)_22_, (GT)_23_, and (GT)_24_, were identified. The (GT)_21_ and (GT)_23_ alleles were significantly enriched among resistant sheep, while (GT)_22_ and (GT)_24_ were more frequent in sensitive animals (χ^2^ = 12.4, *p* = 0.006; Cramér’s V = 0.38). No significant differences in basal *SLC11A1* mRNA expression were detected between phenotypic groups. These findings extend previous GWAS results in sheep by providing the first allele-level evidence linking *SLC11A1* 3′UTR microsatellite polymorphisms to paratuberculosis resistance in sheep. Although limited by sample size and single-breed representation, the results offer a foundation for future functional and genomic selection studies aimed at enhancing disease resilience in small ruminants.

## 1. Introduction

Paratuberculosis (Johne’s disease) is a chronic granulomatous enteritis of ruminants caused by *Mycobacterium avium* subsp. *paratuberculosis* (MAP). The disease leads to persistent diarrhea, weight loss, reduced productivity, and premature culling, resulting in substantial economic losses in livestock industries worldwide [1,2,3,4,5]. Despite its global importance, effective control of paratuberculosis remains challenging due to the pathogen’s long incubation period, subclinical shedding, and incomplete protection conferred by vaccination [6,7,8,9]. Consequently, improving genetic resistance to MAP infection has emerged as a complementary and sustainable control strategy in ruminant breeding programs [10,11,12].

The *solute carrier family 11 member 1* (*SLC11A1*, formerly *NRAMP1*) gene plays a pivotal role in innate immunity by regulating macrophage activation, phagosomal metal transport, and intracellular pathogen control [13,14,15,16,17]. Polymorphisms in *SLC11A1* have been associated with resistance or susceptibility to a variety of intracellular pathogens, including *Mycobacterium tuberculosis*, *Brucella abortus*, and *Salmonella* spp. [18,19,20]. In ruminants, studies in cattle and goats have linked *SLC11A1* variants to differential resistance to paratuberculosis [21,22,23,24]. However, information on this gene’s role in ovine paratuberculosis remains limited and fragmented, despite its potential importance in disease resilience and host–pathogen interactions.

The 3′ untranslated region (3′UTR) of *SLC11A1* contains a highly polymorphic (GT)_n_ microsatellite that has been proposed to affect gene expression through post-transcriptional mechanisms such as modulation of mRNA stability, alteration of secondary structure, and interference with microRNA binding sites [25,26,27]. Variation in this region has been functionally associated with differences in *SLC11A1* transcriptional activity and macrophage response in cattle and goats [21,22]. Nevertheless, the functional and allelic diversity of the 3′UTR (GT)_n_ microsatellite in sheep has not yet been characterized.

In this context, understanding whether variation in the *SLC11A1* 3′UTR (GT)_n_ repeat is associated with MAP resistance could provide valuable insights into the genetic basis of disease resilience in sheep. Such information may complement genome-wide association studies (GWASs), which typically detect SNP-based associations but may overlook functionally important microsatellite polymorphisms [28,29,30,31,32,33,34,35,36].

Therefore, the objective of this study was to investigate the association between allelic variation in the *SLC11A1* 3′UTR (GT)_n_ microsatellite and susceptibility or resistance to paratuberculosis in sheep. By characterizing the allele distribution, gene expression profiles, and their relationship to phenotypic resistance, this study provides the first allele-level evidence for a potential functional role of *SLC11A1* regulatory polymorphisms in ovine paratuberculosis.

## 2. Materials and Methods

### 2.1. Study Design, Population and Sample Collection

The study population comprised 138 adult female sheep (2–5 years old) from three indigenous Greek breeds: Karagouniki (n = 124), Boutsika (n = 5), and Chios (n = 9). The sample size reflects the entire adult female population meeting the health monitoring criteria during the study period. All animals were maintained at the experimental flock of the Agricultural University of Athens, which has been under continuous surveillance for paratuberculosis since 2014 and has not been vaccinated against the disease. The flock is closed, with no introduction of animals from external sources. These breeds were selected because they represent common local genotypes under long-term genomic and health monitoring within our research program [37]. All animals were housed under standard management conditions, with ad libitum access to feed and water. Animals were fed a balanced diet consisting of alfalfa hay, corn, barley, and soybean meal, supplemented with a vitamin–mineral premix formulated according to National Research Council (NRC) guidelines for sheep [38].

To maximize phenotypic contrast animals were classified based on combined diagnostic results obtained over a two-year period (2020–2022) using both ELISA serology and fecal real-time PCR (qPCR) [37,39]. Animals that tested consistently negative by both ELISA (OD% < 20) and qPCR (Ct > 40) across all four semiannual testing rounds were classified as resistant (R, n = 18). Conversely, animals that tested positive by either ELISA (OD% > 40) or qPCR (Ct < 35) in at least three of the four tests were classified as sensitive (S, n = 35). Individuals with inconsistent or borderline results were excluded from the association analysis. As a result, both resistant and sensitive animals originated exclusively from the Karagouniki breed, as none of the Boutsika or Chios sheep met the diagnostic inclusion criteria.

Whole-blood samples were collected aseptically from the jugular vein into heparinized vacutainer tubes, transported to the laboratory within 30 min, and stored at −80 °C until further processing. For RNA analysis, freshly collected blood samples were processed immediately for peripheral blood mononuclear cell (PBMC) isolation.

### 2.2. DNA Extraction and Genotyping of the SLC11A1 3′UTR (GT)_n_ Microsatellite

Genomic DNA was isolated from 200 µL of whole blood using the Nucleospin Tissue DNA kit (Macherey-Nagel GmbH & Co. KG, Düren, Germany) according to the manufacturer’s instructions. Critical for the success of downstream genotyping, DNA quality and quantity were rigorously assessed by 1.5% agarose gel electrophoresis followed by image analysis using a Bio-Rad ChemiDoc XRS+ Molecular Imager (Bio-Rad Laboratories, Inc., Hercules, CA, USA) and absorbance ratio (A260/A280 ratio) using a NanoDrop 8000 spectrophotometer (Thermo Fisher Scientific Inc., Waltham, MA, USA). A further essential quality control step was the confirmation of the absence of PCR inhibitors in all DNA samples by a successful amplification assay targeting the housekeeping gene *β-actin* [40]. Only DNA samples passing all quality controls, demonstrating intact electrophoretic profiles, A260/A280 ratios between 1.8–2.0, and successful *β-actin* amplification, were used for downstream genotyping. DNA samples were stored at −20 °C.

The 3′UTR region of the ovine *SLC11A1* gene (GeneBank: U70255) was amplified via PCR using a Veriti™ Thermal Cycler (Applied Biosystems (Thermo Fisher Scientific), Waltham, MA, USA) for all 138 animals (Table 1). Reactions were prepared in a 25 µL volume containing 1 × KAPA Taq ReadyMix (Kapa Biosystems (a Roche company), Wilmington, MA, USA.), 300 nM of each primer (Forward: 5′-ACCTGGTCTGGACCTGTCTCATCA-3′; Reverse: 5′-CATTGCAAGGTAGGTGTCCCCAT-3′), 2 µL template DNA (≈50 ng), and nuclease-free water. The thermal cycling profile was: initial denaturation at 95 °C for 3 min; 35 cycles of 95 °C for 30 s, 59 °C for 30 s, and 72 °C for 20 s; followed by a final extension at 72 °C for 3 min. The resulting 346 bp amplicons were sequenced on both strands using the BigDye^®^ Terminator Cycle Sequencing Kit and a PRISM^®^ 377 DNA Sequencer (Applied Biosystems (Thermo Fisher Scientific), Waltham, MA, USA).

### 2.3. RNA Extraction and Gene Expression Analysis

Peripheral blood mononuclear cells (PBMCs) were isolated from freshly collected whole blood samples from the 53 animals classified as resistant (R, n = 18) and sensitive (S, n = 35), to preserve cell integrity and RNA quality, preventing degradation, stress-induced gene expression changes, and unwanted cell activation that can distort transcriptomic results. Briefly, 5 mL of heparinized blood was centrifuged at 600× *g* for 15 min, and the buffy coat was diluted 1:1 with PBS–citrate (Sigma-Aldrich, St. Louis, MO, USA) before being carefully layered over 5 mL of Ficoll-Paque (Amersham Biosciences, Uppsala, Sweden). After centrifugation at 500× *g* for 40 min at room temperature with the brake disengaged, the mononuclear cell layer was collected and washed three times in PBS.

Total RNA was extracted from the PBMC pellet using the NucleoSpin^®^ RNA Plus XS Kit, including genomic DNA (gDNA) removal column (Macherey-Nagel GmbH & Co. KG, Düren, Germany), following the manufacturer’s instructions. RNA integrity was confirmed by 1.5% agarose gel electrophoresis followed by image analysis, and purity was verified spectrophotometrically (A260/A280 ≈ 2.0). RNA samples were stored at −80 °C and subjected to no more than one freeze–thaw cycle prior to analysis.

A critical step for gene expression analysis was the thorough elimination of gDNA contamination; therefore, prior to Reverse Transcription-qPCR (RT–qPCR), all RNA samples were further treated with RNase-free DNase I (QIAGEN, Hilden, Germany). The complete removal of gDNA was rigorously verified for each sample by performing no-reverse transcriptase (no-RT) control reactions using *SLC11A1* and *GAPDH* primers (Table 1), which yielded no detectable amplification signal within 40 cycles. As a further essential quality control, the absence of PCR inhibitors was confirmed by the successful amplification of the internal reference gene *GAPDH* in all samples [41].

Relative expression of *SLC11A1* was quantified using the One Step SYBR^®^ PrimeScript™ RT–qPCR Kit II (Takara Bio Inc., Kusatsu, Shiga, Japan) on a LightCycler^®^ 2.0 Real-Time PCR system (Roche Diagnostics GmbH, Mannheim, Germany). Each reaction was prepared to a final volume of 20 µL. For both the *SLC11A1* and the *GAPDH*, the reactions contained 1 × Takara buffer, 400 nM of each primer (*SLC11A1*: Forward: 5′-GGCTGTGGCTGGATTCAAAC-3′; Reverse: 5′-ATGGTCAGCCAGAGGAGAATG-3′ and *GAPDH*: Forward: 5′-TTCCAGTATGATTCCACCCATG-3′; Reverse: 5′-GCCTTTCCATTGATGACGAG-3′), 0.8 µL enzyme mix, 10 ng bovine serum albumin (Thermo Fisher Scientific Inc., Waltham, MA, USA.), 2 µL RNA (≈100 ng total RNA), and RNase-free PCR-grade water.

The thermal profile for *SLC11A1* amplification was: 42 °C for 5 min (reverse transcription); 95 °C for 15 s; 40 cycles of 95 °C for 5 s, 57 °C for 20 s, and 72 °C for 1 s. The profile for *GAPDH* was: 42 °C for 5 min (reverse transcription); 95 °C for 15 s; 40 cycles of 95 °C for 5 s, 52 °C for 20 s, and 72 °C for 1 s. Both protocols concluded with a melting curve analysis (65 °C to 95 °C with a continuous temperature increase at a rate of 0.1 °C/s) and a final cooling step at 40 °C for 30 min.

Amplification specificity was confirmed by melting curve analysis showing a single, sharp peak for both *SLC11A1* and *GAPDH* amplicons. Primer amplification efficiencies were confirmed to be between 90–110% using a standard curve. Each reaction was performed in triplicate, and relative *SLC11A1* mRNA expression was calculated using the 2^−ΔΔCt^ method [42]. Expression values were normalized to *GAPDH*, whose expression stability was verified between groups (*p* > 0.05 for ΔCt comparison by *t*-test), and the mean ΔCt of the sensitive (S) group was used as the calibrator.

### 2.4. Statistical Analysis

Statistical analyses were conducted using GraphPad Prism (v10) and IBM SPSS Statistics (v29.0). A *p*-value of <0.05 was considered statistically significant, unless otherwise stated for multiple testing corrections.

For genotyping data, allele frequencies were calculated as the proportion of individuals carrying each (GT)_n_ repeat allele. Associations between individual allele frequencies and the phenotypic group (resistant vs. sensitive) were evaluated using Fisher’s exact test. A Bonferroni correction for multiple testing was applied to account for the four primary allele comparisons [(GT)_21_, (GT)_22_, (GT)_23_, (GT)_24_], resulting in a corrected significance threshold of α = 0.0125. The overall allele distribution between groups was assessed using a chi-square test of independence. Effect sizes for contingency tests were estimated using Cramér’s V.

For gene expression data, ΔCt values (*SLC11A1* Ct − *GAPDH* Ct) were used for all analyses. Data distribution was assessed for normality using the Shapiro–Wilk test and for homogeneity of variances using Levene’s test. A two-tailed independent samples *t*-test was used to compare ΔCt values between the resistant and sensitive groups. To assess the effect of genotype on gene expression, a one-way analysis of variance (ANOVA) followed by Tukey’s HSD post hoc tests was applied across the different (GT)_n_ genotypes, grouped by the presence of resistance-associated alleles [(GT)_21_ and (GT)_23_] versus sensitivity-associated alleles [(GT)_22_ and (GT)_24_)].

## 3. Results

### 3.1. (GT)_n_ Repeat Polymorphism Frequencies

**Figure 1:**
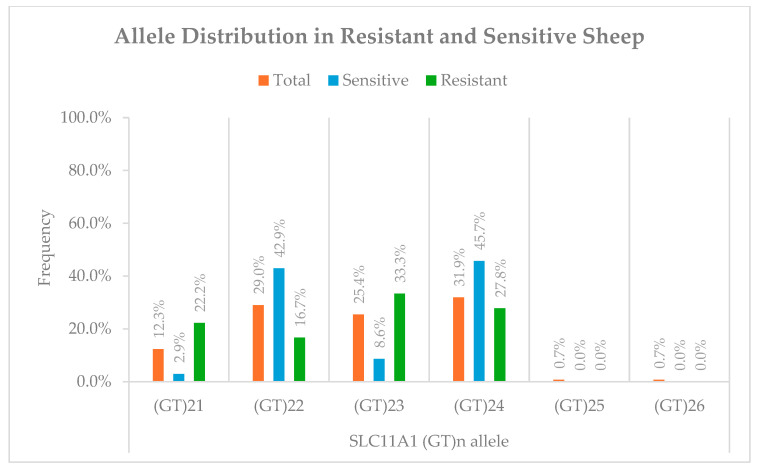
Distribution of ovine *SLC11A1* (GT)_n_ alleles in the study population and in MAP-resistant (R) and MAP-sensitive (S) phenotypic groups.

In the overall population (n = 138), six alleles were identified, corresponding to repeat numbers ranging from 21 to 26. The (GT)_24_ allele was the most frequent, observed in 44 individuals (31.9%), followed by (GT)_22_ in 40 (29.0%), (GT)_23_ in 35 (25.4%), and (GT)_21_ in 17 (12.3%). The rare alleles (GT)_25_ and (GT)_26_ were each found in one individual (0.7%), (Figure 1).

For the genotype–phenotype association analysis, the study focused on the 53 Karagouniki breed with clearly defined resistant (R, n = 18) or sensitive (S, n = 35) status. Within the resistant subgroup, (GT)_23_ was the most frequent allele (33.3%), followed by (GT)_24_ (27.8%), (GT)_21_ (22.2%), and (GT)_22_ (16.7%). In contrast, the sensitive subgroup was dominated by the (GT)_24_ (45.7%) and (GT)_22_ (42.9%) alleles, with (GT)_23_ and (GT)_21_ occurring at much lower frequencies (8.6% and 2.9%, respectively) (Figure 1).

### 3.2. Genotype–Phenotype Association

Fisher’s exact test revealed significant enrichment of the (GT)_21_ allele in resistant sheep (22.2%) compared to sensitive sheep (2.9%) (uncorrected *p* = 0.040, OR = 9.5, 95% CI: 1.00–89.5). Similarly, (GT)_23_ was more frequent in resistant animals (33.3%) than in sensitive ones (8.6%) (uncorrected *p* = 0.048, OR = 5.3, 95% CI: 1.00–28.7). However, after applying a Bonferroni correction for the four primary allele comparisons (corrected significance threshold α = 0.0125), these individual associations were no longer statistically significant.

Conversely, the (GT)_24_ and (GT)_22_ alleles were more prevalent among sensitive sheep (45.7% and 42.9%, respectively) than resistant sheep (27.8% and 16.7%, respectively), though these differences did not reach statistical significance (*p* = 0.217 and *p* = 0.063, respectively).

When allele distributions were analyzed overall, a significant association was observed between genotype and phenotype (χ^2^ = 12.4, df = 3, *p* = 0.006), with Cramér’s V = 0.38, indicating a moderate effect size.

To place our findings in context, Table 2 summarizes reported associations between *SLC11A1* polymorphisms and resistance/susceptibility across species.

### 3.3. SLC11A1 Gene Expression

**Figure 2:**
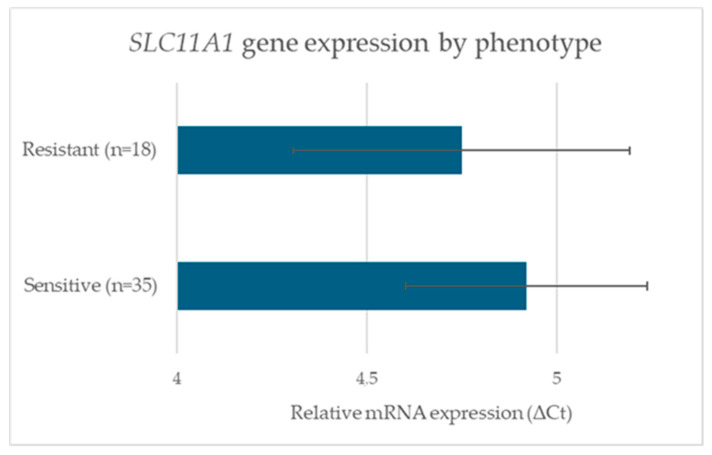
Comparison of *SLC11A1* gene expression levels between MAP-resistant and MAP-sensitive sheep. Relative mRNA expression was quantified by RT-qPCR and reported as ΔCt values (normalized to *GAPDH*). Bars show the mean ΔCt for each group (Sensitive, n = 35; Resistant, n = 18). Error bars indicate the 95% confidence interval of the mean (Resistant: 4.29–5.21; Sensitive: 4.60–5.24). An independent samples *t*-test found no statistically significant difference between groups (t(51) = 0.63, *p* = 0.531, Cohen’s d = 0.17).

An independent-samples two-tailed *t*-test showed no significant difference between groups [mean ΔCt_S = 4.92 ± 0.84, mean ΔCt_R = 4.75 ± 0.91; t(51) = 0.63, *p* = 0.531; 95% CI for the difference: −0.39 to 0.73; Cohen’s d = 0.17] (Figure 2). A one-way ANOVA comparing expression across (GT)_n_ genotype groups likewise found no significant effect [F(2,50) = 1.42, *p* = 0.25; η^2^ = 0.05]. Post hoc pairwise comparisons using Tukey’s HSD confirmed the absence of significant differences between genotypic categories.

## 4. Discussion

This study provides evidence that variation in the 3′UTR (GT)_n_ microsatellite of the *SLC11A1* gene is associated with resistance to natural MAP infection in sheep. Six allelic variants were identified within the 138 animals examined, with four alleles (GT)_21_, (GT)_22_, (GT)_23_, and (GT)_24_ being common in the study population. Among these, the (GT)_21_ and (GT)_23_ alleles appeared more frequently in sheep classified as resistant, whereas (GT)_22_ and (GT)_24_ were more frequent among sensitive animals. Although individual allele comparisons did not retain statistical significance after Bonferroni correction, the overall allelic distribution differed significantly between resistant and sensitive groups (*p* = 0.006), suggesting that the *SLC11A1* 3′UTR microsatellite may contribute to host resistance to MAP infection in sheep. The moderate effect size (Cramér’s V = 0.38) further supports the biological relevance of this association, despite the statistical limitations imposed by the sample size. These results are consistent with previous findings in goats and cattle, where variation in the *SLC11A1* 3′UTR microsatellite has been associated with altered resistance to bacterial infections, including paratuberculosis and brucellosis [21,23,24,25,43] (Table 2). The absence of significant differences in *SLC11A1* mRNA expression between resistant and sensitive animals under basal conditions indicates that microsatellite variation does not strongly influence transcriptional levels in unstimulated PBMCs [22,23]. However, the possibility remains that such polymorphisms modulate expression upon immune stimulation, as demonstrated in goats and cattle following MAP challenge [22,23,49]. This dissociation between genotype and basal expression suggests a condition-dependent regulatory mechanism [21,22]. The (GT)_n_ repeat may alter the secondary structure of the 3′UTR, affecting its interaction with microRNAs or RNA-binding proteins that regulate mRNA stability and translation specifically during immune responses [21,25]. Thus, constitutive *SLC11A1* expression may not differ markedly between phenotypic groups, but the allelic context could still affect inducible expression during infection or cytokine stimulation, a hypothesis that merits further investigation using MAP-infected macrophages.

Importantly, this study provides one of the first characterizations of the *SLC11A1* 3′UTR microsatellite in sheep. Previous GWASs in this species have implicated regions on chromosomes 2, 3, and 20 containing immune-related genes, including *SLC11A1* [33,34]. However, most GWASs rely on SNP-based analyses that may not capture functional variation in microsatellite loci [50]. The current findings therefore add complementary evidence by resolving a specific, functional polymorphism within a broader GWAS-implicated region, highlighting the potential importance of non-coding repetitive elements in host–pathogen interactions.

A limitation of the present work is the relatively small number of resistant animals (n = 18), which restricts statistical power and may increase the risk of Type II errors. Nevertheless, the study flock represents a closed population that has been maintained without external animal introductions since its establishment, under consistent management and monitored conditions. This closed design ensures uniform environmental exposure and infection pressure, minimizing confounding factors related to management or pathogen circulation [7,51,52]. Moreover, the flock has been monitored longitudinally for over a decade, enabling robust phenotypic classification based on repeated diagnostic testing. Both resistant and sensitive groups comprised exclusively Karagouniki sheep, as positive and negative diagnostic results were available only for this breed, precluding breed-stratified analyses. Future research including a broader range of breeds and larger sample sizes would enable assessment of potential breed-specific linkage disequilibrium patterns, as previously suggested by GWAS analyses [34,53].

Collectively, the present results provide evidence that variation in the *SLC11A1* 3′UTR microsatellite may contribute to differential resistance to paratuberculosis in sheep. Although individual allele associations did not remain significant after multiple testing correction, the observed trend aligns with prior functional studies in other ruminants, indicating a conserved regulatory mechanism influencing disease susceptibility. These findings position the *SLC11A1* microsatellite as a candidate locus for marker-assisted selection. Expanding this research to larger, multi-breed cohorts and evaluating inducible *SLC11A1* expression under infection conditions will be essential to establish the functional relevance of these microsatellite variants and assess their practical utility in breeding programs aimed at sustainable paratuberculosis control.

## 5. Conclusions

This study provides the first preliminary evidence that polymorphisms in the 3′UTR (GT)_n_ microsatellite of the *SLC11A1* gene are associated with resistance to natural MAP infection in sheep. A significant overall difference in allele distribution was observed between resistant and sensitive animals, with the (GT)_21_ and (GT)_23_ alleles emerging as potential resistance markers, while (GT)_22_ and (GT)_24_ were more frequent among sensitive individuals. Although individual allele associations did not remain significant after strict correction for multiple testing, the strength of the overall association and the moderate effect size indicate a biologically meaningful trend that merits further investigation. The absence of significant differences in basal *SLC11A1* mRNA expression in PBMCs suggests that the microsatellite does not markedly influence constitutive transcription but may affect post-transcriptional regulation, potentially altering mRNA stability or translation efficiency upon immune activation.

While the relatively small number of resistant animals and the single-breed design limit broad generalization, this work establishes a foundation for further research into the regulatory role of *SLC11A1* 3′UTR variation in inducible immune responses. Future studies encompassing multiple breeds, larger cohorts, and integrated analyses of SNP and microsatellite variation together with gene expression under stimulation or infection will be essential to validate these associations and evaluate their potential utility in marker-assisted breeding strategies aimed at enhancing paratuberculosis resistance in sheep populations.

## Figures and Tables

**Table 1:** The primer sequences, product sizes, cycling conditions and relevant references for the assays targeting *IS900*, *SLC11A1* 3′UTR, *GAPDH*, *SLC11A1* mRNA, and *β-actin* used in this study.

Target	Primers (5′-3′)	Size	Thermal Profile	Reference
*IS900*	F ^1^: AATGACGGTTACGGAGGTGGT R ^2^: GCAGTAATGGTCGGCCTTACC Pr ^3^: TCCACGCCCGCCCAGACAGG	76 bp	95 °C for 3 min; 40 cycles of 95 °C for 3 s, 60 °C for 20 s, 72 °C for 1 s; 43 °C for 30 s	[39]
3′UTR *SLC11A1*	F: ACCTGGTCTGGACCTGTCTCATCA R: CATTGCAAGGTAGGTGTCCCCAT	346 bp	95 °C for 3 min; 35 cycles of 95 °C for 30 s, 59 °C for 30 s, 72 °C for 20 s; 72 °C for 3 min	[23]
*GAPDH*	F: TTCCAGTATGATTCCACCCATG R: GCCTTTCCATTGATGACGAG	80 bp	42 °C for 5 min; 95 °C for 15 s; 40 cycles of 95 °C for 5 s, 52 °C for 20 s, 72 °C for 1 s; 40 °C for 30 s	[41]
*SLC11A1* mRNA	F: GGCTGTGGCTGGATTCAAAC R: ATGGTCAGCCAGAGGAGAATG	168 bp	42 °C for 5 min; 95 °C for 15 s; 40 cycles of 95 °C for 5 s, 57 °C for 20 s, 72 °C for 1 s; 40 °C for 30 s	[23]
*β-actin*	F: TGTCTCTGTACGCTTCTGG R: GTGGTGGTGAAACTGTAGC	190 bp	95 °C for 3 min; 40 cycles of 95 °C for 30 s, 55 °C for 30 s, 72 °C for 30 s; 72 °C for 3 min	[40]

^1^ Forward; ^2^ Reverse; ^3^ Probe.

**Table 2:** Comparative summary of reported associations between *SLC11A1* polymorphisms and disease-related phenotypes in livestock and humans.

Species	Variant/Region Analyzed	Association with Resistance/Susceptibility	Notes	References
Sheep	Genetic influences (preliminary, candidate-based)	Suggested possible genetic effect on Johne’s disease susceptibility	Early evidence, not locus-specific	[11]
Sheep	Retrospective SNP analysis	Identified associations near *SLC11A1* with MAP resistance	Based on FFPE DNA, SNP focus	[27]
Sheep	GWAS (antibody response to MAP)	Regions linked to immune response; *SLC11A1* implicated	High-resolution genomic mapping	[33]
Sheep	GWAS (SNPs across genome)	Regions associated with MAP resistance; included *SLC11A1*	SNP-based, no microsatellite resolution	[34]
Sheep	3′UTR (GT)_n_ microsatellite	(GT)_21_ and (GT)_23_ associated with resistance; (GT)_22_ and (GT)_24_ with susceptibility	Association found despite no difference in basal expression	[This study]
Goats	Functional analysis, 3′UTR microsatellite	Variants affected inducible expression under MAP challenge	Demonstrated functional mechanism	[21]
Goats	3′UTR (GT)_n_ microsatellite	Shorter alleles enriched in resistant goats	Consistent with ovine findings	[23]
Goats	3′UTR microsatellite	Specific alleles associated with reduced paratuberculosis incidence	Validated earlier results	[24]
Cattle	SNPs in *SLC11A1*	Associated with MAP infection risk	Consistent across populations	[26]
Cattle	Candidate gene SNPs (*SLC11A1*, *TLR4*, *IFNG*)	Associations with MAP susceptibility	Population-specific variation	[43]
Cattle	SNPs in *SLC11A1* and others	Linked with breeding values for MAP traits	Large-scale genomic approach	[44]
Cattle	SNPs in *SLC11A1*	No association with MAP infection	SNPs polymorphic variants showed no allele/genotype differences between cattle	[45]
Cattle	*SLC11A1* SNP rs109453173	Associated with resistance (GG genotype/G allele protective; CC/CG linked to susceptibility)	Case–control study; suggests potential resistance marker	[46]
Buffalo	3′UTR microsatellite	Allelic variation influenced *MCP1* mRNA after *Brucella* challenge	Functional immune effects	[25]
Pigs	*SLC11A1* polymorphisms	Associated with immune traits	Cross-species evidence of functional role	[47]
Humans	*SLC11A1* SNPs and promoter variants	Associated with tuberculosis susceptibility	Strong parallels with livestock	[18,48]

## Data Availability

The original contributions presented in this study are included in the article. Further inquiries can be directed to the corresponding author(s).
